# Estimation of the Total Number of SARS-CoV-2-infected Individuals and the Necessary Tests and Cost During the First Wave of the COVID-19 Pandemic in Japan

**DOI:** 10.2188/jea.JE20210197

**Published:** 2021-10-05

**Authors:** Nobutoshi Nawa, Daiki Tebi, Jin Kuramochi, Takeo Fujiwara

**Affiliations:** 1 Department of Medical Education Research and Development, Tokyo Medical and Dental University, Tokyo, Japan; 2 Department of Global Health Promotion, Tokyo Medical and Dental University, Tokyo, Japan; 3 Kuramochi Clinic Interpark, Utsunomiya, Tochigi, Japan

Dear Editor,

The number of reverse transcription polymerase chain reaction (RT-PCR) tests for severe acute respiratory syndrome coronavirus 2 (SARS-CoV-2) in Japan was small (ie, 26 tests per million, as compared to 196 tests per million in South Korea).^1^ Given the characteristics of SARS-CoV-2, which involves pre-symptomatic transmission,^2^^,^^3^ a shortage of testing makes it difficult to understand the precise situation of the pandemic.^4^ As a result, the implementation of widespread testing has been proposed.^5^^–^^7^ However, no study has estimated the ideal testing capacity based on epidemiological data using SARS-CoV-2 serological study results. To that end, we aimed to calculate the total number of tests necessary to capture every individual infected with SARS-CoV-2 in the first wave of the pandemic in Japan.

We defined a “case” as an individual whose RT-PCR test report for SARS-CoV-2 was positive. We used an open dataset provided by Toyo Keizai Online^8^ to calculate the total number of cases and individuals tested from February 8 through May 25, 2020 (ie, the first wave of the pandemic). We also used data from the Utsunomiya COVID-19 seROprevalence Neighborhood Association (U-CORONA) study.^9^ Utsunomiya city was chosen because it is close to Tokyo, the prefecture with the highest number of infected people, and is home to both elderly and child-rearing generations. We estimated the prevalence in each age group (<15, 15–64, and >64 years old) for each prefecture (eMaterials 1).

We calculated the total number of infected individuals based on the estimated prevalence of SARS-CoV-2 by age group in Utsunomiya City. We multiplied this estimated prevalence by the number of inhabitants in each prefecture by the age group^10^ and summed them to estimate total number of infected individuals. The number of necessary RT-PCR tests was calculated by dividing the estimated number of infected individuals by the mean positive rate among individuals who underwent testing in each prefecture. The cost of the tests was estimated by multiplying the number tests required by the cost per test.^11^

Scatter plots of the testing rate per population and the positive rate from February 8 through May 5, 2020 (ie, in the first wave) and the estimated numbers of cases in each prefecture are presented in eTable 1, eFigure 1, and eMaterials 2. Table 1 presents the estimated prevalence, the total number of infected individuals, and the number of necessary tests. The estimated overall prevalence was 1.23%. The estimated total number of infected individuals was 1,547,280. The total number of required tests was 44,590,196, which is equal to 35.3% of the population of Japan. The cost of the estimated total number of required tests is JPY 802,623,528,000 (∼USD 7.7 billion).

**Table 1.** Estimated prevalence, total number of infected individuals, number of required tests, and cost of testing until the state of emergency was lifted for each prefecture, stratified by age group

Age, years	Population	Prevalence, %	Total number of infected individuals	Number of required tests	Cost of testing, JPY
<15	15,213,000	2.22	337,729	9,732,813	175,190,634,000
15–64	75,074,000	1.31	983,469	28,342,058	510,157,044,000
>64	35,886,000	0.63	226,082	6,515,326	117,275,868,000
Total	126,173,000	1.23	1,547,280^*^	44,590,196	802,623,528,000

JPY, Japanese yen.

^*^The total number of infected individuals was calculated by summing the numbers in each age group. Thus, this value was different from the product of the total population (126,173,000) and overall prevalence (1.23%).

The estimated number of RT-PCR tests required to capture all the individuals infected with SARS-CoV-2 in the study period was about 600 times that of today’s capacity to conduct RT-PCR test in Japan, suggesting it would take almost 1 year and 7 months to complete.^12^ This would be infeasible. Our result also suggests that there were many more infected individuals in the community than reported.

Our study has limitation. We extrapolated the estimated prevalence of SARS-CoV-2 in Utsunomiya City to all of Japan with only an age adjustment based on an age variable with three categories. In future studies, factors besides age—such as industry, mask use, and social-gathering prevalence—must be considered. These discussions are presented in more detail in eMaterials 3.

In conclusion, our result suggests that there are many more infected individuals in the community than reported in the first wave, and to test them all would require about 600 times the current capacity of RT-PCR testing in Japan. This would be infeasible at the current capacity. These figures may be useful for discussing the testing practices needed to address COVID-19 in Japan.

## APPENDIX A. SUPPLEMENTARY DATA

eMaterials 1. Detailed description of prevalence estimation using data from the U-CORONA Study

eTable 1. Total number of infected individuals, number of required tests, and cost of testing until the state of emergency was lifted for each prefecture, stratified by prefecture

eFigure 1. Relationship between testing rate per population and positive rate until the state of emergency was lifted in each prefecture

eMaterials 2. More description for eFigure 1

eMaterials 3. More discussion of the limitations
