# Supplementary material for: Estimation of the Total Number of SARS-CoV-2-infected Individuals and the Necessary Tests and Cost During the First Wave of the COVID-19 Pandemic in Japan
Source: J Epidemiol. 2021 Oct 5;31(10):554–5. doi: 10.2188/jea.JE20210197 (PMC8421198; doi:10.2188/jea.JE20210197)
Supplement: Supplementary file 1 [file je-31-554-s001.pdf]

eMaterials 1. Detailed description of prevalence estimation using data from the U-CORONA Study

Prevalence estimates were obtained from the U-CORONA Study, as described previously.[9] Briefly, multiple imputation was used to estimate the population-based weighted prevalence according to baseline data from the registry, which included age, sex, distance to clinic, residential district, and number of cohabitants of participants and non-participants. The analysis was performed in STATA 14 (StataCorp LP, College Station, TX, USA).

eTable 1. Total number of infected individuals, number of required tests, and cost of testing until the state of emergency was lifted for each prefecture, stratified by prefecture

Prefecture	Prevalence, %	Total number of infected individuals	Number of required tests	Cost of testing, JPY
**Hokkaido**	1.19	62,540	1,802,308	32,441,544,000
**Aomori**	1.18	14,724	424,323	7,637,814,000
**Iwate**	1.19	14,560	419,585	7,552,530,000
**Miyagi**	1.23	28,250	814,127	14,654,286,000
**Akita**	1.15	11,078	319,248	5,746,464,000
**Yamagata**	1.19	12,780	368,300	6,629,400,000
**Fukushima**	1.2	22,145	638,187	11,487,366,000
**Ibaraki**	1.22	34,859	1,004,579	18,082,422,000
**Tochigi**	1.23	23,720	683,568	12,304,224,000
**Gunma**	1.22	23,620	680,706	12,252,708,000
**Saitama**	1.24	90,967	2,621,536	47,187,648,000
**Chiba**	1.23	76,865	2,215,141	39,872,538,000

**Tokyo**	1.25	174,663	5,033,519	90,603,342,000
**Kanagawa**	1.25	114,671	3,304,628	59,483,304,000
**Niigata**	1.19	26,537	764,746	13,765,428,000
**Toyama**	1.19	12,455	358,922	6,460,596,000
**Ishikawa**	1.22	13,890	400,294	7,205,292,000
**Fukui**	1.22	9,346	269,323	4,847,814,000
**Yamanashi**	1.21	9,789	282,092	5,077,656,000
**Nagano**	1.2	24,676	711,138	12,800,484,000
**Gifu**	1.22	24,213	697,790	12,560,220,000
**Shizuoka**	1.22	44,386	1,279,130	23,024,340,000
**Aichi**	1.26	95,084	2,740,164	49,322,952,000
**Mie**	1.22	21,724	626,052	11,268,936,000
**Shiga**	1.26	17,809	513,216	9,237,888,000
**Kyoto**	1.22	31,438	905,988	16,307,784,000
**Osaka**	1.23	108,351	3,122,510	56,205,180,000
**Hyogo**	1.22	66,932	1,928,885	34,719,930,000

**Nara**	1.2	16,038	462,199	8,319,582,000
**Wakayama**	1.19	10,997	316,925	5,704,650,000
**Tottori**	1.21	6,710	193,378	3,480,804,000
**Shimane**	1.19	8,014	230,948	4,157,064,000
**Okayama**	1.22	23,019	663,380	11,940,840,000
**Hiroshima**	1.23	34,394	991,176	17,841,168,000
**Yamaguchi**	1.18	16,072	463,167	8,337,006,000
**Tokushima**	1.18	8,608	248,066	4,465,188,000
**Kagawa**	1.2	11,527	332,202	5,979,636,000
**Ehime**	1.19	15,995	460,960	8,297,280,000
**Kochi**	1.17	8,172	235,496	4,238,928,000
**Fukuoka**	1.24	63,256	1,822,948	32,813,064,000
**Saga**	1.23	9,992	287,942	5,182,956,000
**Nagasaki**	1.2	15,955	459,798	8,276,364,000
**Kumamoto**	1.22	21,327	614,602	11,062,836,000
**Oita**	1.2	13,597	391,844	7,053,192,000

**Miyazaki**	1.21	13,005	374,778	6,746,004,000
**Kagoshima**	1.21	19,456	560,692	10,092,456,000
**Okinawa**	1.31	19,074	549,689	9,894,402,000

JPY, Japanese yen.

eFigure 1. Relationship between testing rate per population and positive rate until the state of emergency was lifted in each prefecture

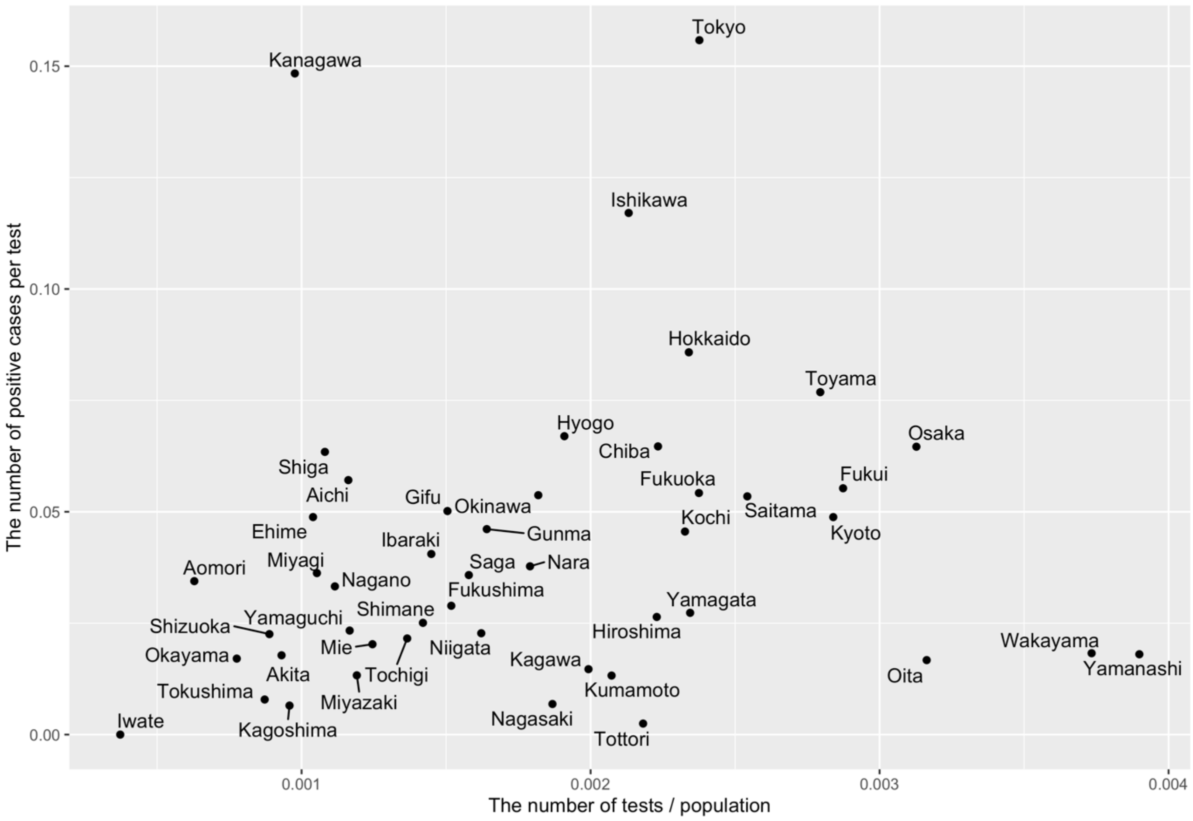

The data source for this figure was provided by Toyo Keizai Online[8] and Ministry of Internal Affairs and Communications.[10] Detailed explanations related to the figure, including how the values in the plot are calculated, can be found in **eMaterials 2**.

eMaterials 2. More description for eFigure 1

To determine the association between the number of individuals who underwent reverse transcription polymerase chain reaction (RT-PCR) testing and the rate of positive cases, we created a scatter plot of the RT-PCR testing rate per prefectural population (number of individuals who were tested divided by the population of each prefecture) and rate of positive cases (number of individuals who tested positive divided by the number of individuals who were tested in each prefecture) up to the end of the state of emergency in each prefecture, using data provided by Toyo Keizai Online[8] and the population data for each prefecture.[10] The number of RT-PCR tests in Japan was limited and testing was highly selective, especially at the beginning of the pandemic. We assumed that healthcare professionals tested individuals who were most likely to be infected on a priority basis. Therefore, during the first wave, the positive rate was likely to be the highest and would not have increased along with the number of tests. From the plot in **eFigure 1**, we estimated the maximum positive rate in Japan using the mean positive rate.

The testing rate per population ranged from 0.037% to 0.39%, while the positive rate ranged from 0.0% to 15.6%. Three prefectures (i.e., Tokyo, Kanagawa, and Ishikawa) were considered outliers; there, the positive rate was substantially higher than that in other prefectures. Thus, we excluded these prefectures from the estimation of the mean rate of positive cases. This rate was not associated with the testing rate ($r=0.27$, $p=0.08$), and the

mean rate of positive cases excluding outliers (i.e., Ishikawa, Tokyo, and Kanagawa) was 3.47% (standard deviation, 2.12).

eMaterials 3. More discussion of the limitations

In this study, the number of necessary reverse transcript polymerase chain reaction (RT-PCR) tests was calculated by dividing the estimated number of infected individuals by the mean positive rate, which we assumed not to change as the number of infected individuals increased. This was based on the observation that the positive rate was not associated with the testing rate in our data (**eFigure 1**). This assumption may not hold if the positive rate decreases as the number of tests increases;[4] however, if this were the case, our estimate would represent the minimum number of tests required. Thus, we believe that these figures may be useful for discussing the testing practices needed to address COVID-19 in Japan. Finally, while the infection status during the study period varied greatly from region to region, we assumed this status to be constant throughout Japan. We believe that it is essential for the effective discussion of countermeasure policies to have a clear target for the number of tests required. However, no study to date has estimated the ideal testing capacity corresponding to the scale of the pandemic based on epidemiological data; thus, in this research, we sought to calculate the total number of tests necessary to capture every individual infected with SARS-CoV-2 in the first wave of the pandemic in Japan.
